# Evaluating the Vaccine Potential of a Tetravalent Fusion Protein (*rBm*HAXT) Vaccine Antigen Against Lymphatic Filariasis in a Mouse Model

**DOI:** 10.3389/fimmu.2018.01520

**Published:** 2018-07-02

**Authors:** Nikhil Chauhan, Vishal Khatri, Priyankana Banerjee, Ramaswamy Kalyanasundaram

**Affiliations:** Department of Biomedical Sciences, University of Illinois, Rockford, IL, United States

**Keywords:** lymphatic filariasis, TLR-4, prophylactic vaccine, aluminum, multivalent antigen, mouse model

## Abstract

Lymphatic filariasis (LF) is a tropical parasitic infection of human transmitted by mosquitoes. Chronic infection results in severe physical disability in the infected patients. Although several potential vaccine antigens were identified by several groups, there are no licensed prophylactic vaccine to date against this infection in the human. Previous attempts from our laboratory to develop a trivalent prophylactic vaccine against LF showed that >90% protection could be achieved in rodent models. However, this trivalent vaccine gave only 35% protection in non-human primates. The major focus of this study was to develop a tetravalent prophylactic vaccine (r*Bm*HAXT) and test the vaccine potential in a mouse model. We evaluated three different adjuvant formulations; alum, glucopyranosyl lipid adjuvant in stable emulsion (GLA/SE) alum (AL019), and mannosylated chitosan (MCA) to determine the optimum adjuvant formulation for r*Bm*HAXT. Results presented in this study show that r*Bm*HAXT + AL019 gave the highest rate of protection (>88%) against challenge infection, compared to r*Bm*HAXT + AL007 (79%), r*Bm*HAXT + MCA (79%) and controls. Analysis of the immune correlates of protection showed that all three adjuvants elicited high titer of antigen-specific IgG1, IgG2a, and IgG2b antibodies. High number of IFN-γ-producing antigen-specific memory cells were generated in the vaccinated animals irrespective of the adjuvants used. Similarly, spleen cells from r*Bm*HAXT-vaccinated animals secreted IL-4, IL-10, and IFN-γ in response to r*Bm*HAXT suggesting the generation of a balanced Th1/Th2 response. There was also an increase in IL-17-secreting cells in r*Bm*HAXT-vaccinated animals. These findings thus suggest that r*Bm*HAXT + AL019 is a better prophylactic formulation for LF.

## Introduction

Lymphatic filariasis (LF) is a neglected tropical parasitic disease caused by three filarial parasites, *Wuchereria bancrofti, Brugia malayi*, and *Brugia timori* and is transmitted by mosquitoes. According to the World Health Organization, currently 856 million people residing in 52 countries require preventive chemotherapy to stop the spread of infection (1–4). As a preventive strategy, The Global Program to Eliminate LF was launched in 2000 to eradicate LF by 2020 using Mass Drug Administration (MDA) with diethylcarbamazine (6 mg/kg) and albendazole (400 mg) (3). So far, preventive chemotherapy is the only option to stop the spread of LF infection in endemic regions. Ten countries (Egypt, Cambodia, Cook Islands, Maldives, Marshall Islands, Niue, Sri Lanka, Thailand, Togo, and Vanuatu) have to date declared achieving elimination of LF (4). However, there have been few reports of reemergence of LF infection in Sri Lanka (5). Preventive chemotherapy is still needed in all other countries endemic for this disease and has not achieved the desired levels of coverage as of 2017 (6, 7). As in Sri Lanka, there is fear of reemergence of the infection in areas where MDA was given, mainly due to subject noncompliance (8–11). Even one infected individual left untreated in a community can be disastrous because drugs can only treat current infection and will not prevent future infections, especially once the drug effect wanes from the system. Therefore, there is a need for a more sustainable approach to control and eliminate the infection. Prophylactic vaccination with MDA will be a more sustainable and long lasting approach to eliminate infection from a community by eliciting herd immunity (2).

Several potential vaccine candidates were identified from our laboratory and others (12–21) were shown to confer varying degrees of protection against challenge infection with *B. malayi* in experimental animals. Out of the several candidate vaccine antigens reported to date, we selected three best candidate antigens; heat shock protein 12.6 (HSP-12.6) (22), abundant larval transcript-2 (ALT-2) (21), and tetraspanin large extracellular loop (TSPLEL) (23) that repeatedly showed maximum protection in rodent models in our laboratory and other. Two of the selected antigens were then combined with a bivalent vaccine antigen and tested the vaccine potential in rodent models (24–28). Subsequently, we combined three of the antigens with a trivalent fusion protein vaccine and tested in rodents (29–33) and in non-human primates (34). The trivalent vaccine gave excellent protection against challenge infections in the rodent models (~95% protection). However, the trivalent vaccine (r*Bm*HAT) when given along with alum gave only ~35% protection in non-human primates (34). The immune responses generated following the vaccination in non-human primates was predominantly Th2 biased and this was attributed to the poor protective responses. Protection against LF in the human and rhesus macaques is correlated with a balanced Th1/Th2 response (29, 35, 36). Therefore, the major focus of this study was to construct a tetravalent vaccine (r*Bm*HAXT) by adding another antigen, thioredoxin peroxide (*Bm*TPX-2) that is known to promote a Th1 response, to the trivalent vaccine and evaluate its vaccine potential in a mouse model. Thioredoxin peroxidase (TPX-2) is a highly immunogenic protein within the total secretome of *B. malayi* (37–39). Several groups including ours have reported the vaccine potential of TPX-2 previously (24, 27, 28, 40–42). In fact, one of our studies showed that a trivalent fusion protein *rBm*HAX (HSP12.6 + ALT-2 + TPX-2) vaccine conferred 67.5% protection in the mouse model (30). Immunization with TPX-2 skewed the protective immune response to a Th1 bias (30, 41, 43). Therefore, we hypothesized that a tetravalent fusion protein could generate a more balanced Th1/Th2 response and will be more effective as a prophylactic vaccine against LF infection. In this study, we also evaluated the potential of three different adjuvant formulations [alum (AL007), glucopyranosyl lipid adjuvant in stable emulsion (GLA-SE)/alum (a synthetic toll-like receptor 4 agonist on alhydrogel, AL019), and mannosylated chitosan (MCA)] for their ability to promote a balanced Th1/Th2 response when given along with the tetravalent r*Bm*HAXT vaccine. One of our previous studies showed that AL019 and MCA are excellent adjuvants for the trivalent r*Bm*HAT vaccine antigen and promoted significant vaccine-induced protection in the mouse model (30).

Thus, the major focus of this study was to evaluate the vaccine potential of a tetravalent fusion protein antigen, r*Bm*HAXT against *B. malayi* challenge infections in a mouse model.

## Materials and Methods

### Construction of Tetravalent Coding Sequence

Multivalent gene sequences of *bmhaxt* consisting of *bmhsp12.6* (accession #AY692227.1), *bmalt-2* (accession #JF795950.1), *bmtpx-2* (accession #AF319997.1), and *bmtsp* (accession #JF795955.1) was constructed at Genscript (Piscataway, NJ, USA).

### Cloning, Expression, and Purification of *rBm*HAXT Recombinant Protein

GenScript supplied the sequences in the pUC57 vector. The genes were amplified using forward CG**GGATCC**ATGGAAGAAAAGGTAGTG and reverse CCC**GAATTC**TTAATGTTTCTCAAAATATGCTTT primers with restriction sites for *BamHI* and *EcoRI*. The PCR-amplified products were cloned into the pRSETA expression vector, transformed into competent BL21 (DE3) *Escherichia coli* cells for expression of the recombinant proteins with 6× histidine tag. Recombinant fusion proteins were purified using immobilized metal affinity Ni^+^-charged Sepharose column (GE Healthcare Life Sciences, Pittsburg, PA) and eluted with 300 mM imidazole. Endotoxin in the final purified protein preparation was removed using an endotoxin removal column (Thermo Fisher Scientific, Rockford, IL, USA). The expression and purity of recombinant proteins was confirmed in 12% SDS-PAGE gel and Western blot using anti-His antibodies (Qiagen, Valencia, CA, USA). Protein concentration was determined using a Bradford reagent (Thermo Fisher Scientific).

### Adjuvants

We used three different adjuvant formulations with recombinant *Bm*HAXT. Alum (AL007) and Alum plus GLA, a synthetic TLR4 agonist (AL019) was purchased from the Infectious Disease Research Institute, Seattle, WA and MCA was a gift from Pacific GeneTech, Hong Kong.

### Animals and Parasite

Six- to eight-week-old BALB/c mice purchased from Taconic biosciences (Hudson, NY, USA) were used in these experiments. Use of animal in this study was approved by the animal care committee of the University of Illinois, Rockford following the National Institutes of Health guidelines for the care and use of laboratory animals. The infective larval stage (L3) of *B. malayi* was obtained from the NIAID/NIH Filariasis Research Reagent Resource Center (University of Georgia, Athens, GA, USA).

### Immunization of BALB/c Mice

For the immunization, mice were randomly divided into seven groups of five mice each per group as described below: (1) r*Bm*HAXT + AL007 given s/c, (2) r*Bm*HAXT + AL019 given s/c, (3) r*Bm*HAXT + MCA (first dose s/c and booster doses given orally), (4) AL007 control given s/c, (5) AL019 control given s/c, (6) MCA control (first dose s/c and booster doses given orally), and (7) r*Bm*HAXT + MCA control (all doses were given orally). Each mouse received three doses of 15 µg of r*Bm*HAXT and 15 µg of respective adjuvant formulation at 15 days interval.

### Collection of Serum, Peritoneal Fluid, and Spleen

Blood samples were collected from the submandibular vein of each mouse on day 0 (pre-immune), and then 2 weeks after each immunization and kept at room temperature for 1 h to clot. Serum was separated, and aliquots were kept frozen at −80°C for further use. Peritoneal cavity was washed with 500 µl of sterile saline solution and the fluid was collected from each mouse and processed. Spleen was then collected from each animal, washed three times with complete RPMI-1640 medium supplemented with 10% FBS and 1 × antibiotic/mycotic solution (Sigma, St. Louis, MO, USA).

### Determining the Antibody Titer by ELISA

#### Titer of IgG Antibodies in the Serum and Peritoneal Fluids

The titer of r*Bm*HAXT-specific IgG antibodies in the sera samples and in the peritoneal fluids were evaluated using an indirect ELISA as described previously. Wells were coated with 1 µg/ml of r*Bm*HAXT overnight at 4°C. After washing and blocking the plates, diluted (1:100, 1:1,000, 1:5,000, 1:10,000, 1:20,000, and 1:40,000) sera or peritoneal fluid samples were added and incubated for 1 h at room temperature. HRP-conjugated chicken anti-mouse IgG antibodies (Thermo Fisher scientific) were used as the secondary antibodies and color was developed using the 1-step Ultra TMB-ELISA substrate (Thermo Fisher Scientific). The reaction was stopped using 0.16 M H_2_SO_4_, and optical density was determined at 450 nm in a BioTek Synergy 2 ELISA reader.

### Levels of Antigen-Specific Antibody Isotypes in the Serum and Peritoneal Fluids

Levels of r*Bm*HAXT-specific antibody isotypes (IgG1, IgG2a, IgG2b, IgG3, IgE, IgM and IgA) were determined in the sera and peritoneal fluid samples using an indirect ELISA as described above. Respective isotype-specific biotinylated goat anti-mouse antibodies (Sigma) and streptavidin-HRP (1:20,000) were used as the secondary antibodies. Color was developed with 1-step Ultra-TMB. The reaction was stopped using 0.16 M H_2_SO_4_ and optical density was determined at 450 nm in a BioTek Synergy 2 ELISA reader.

### Challenge Studies

To determine the vaccine-induced protection, we used a micropore chamber challenge method as described previously (29–33). Briefly, 20 L3s of *B. malayi* were placed in a micropore chamber and surgically implanted into the peritoneum of each mouse. Following, 72 h implantation, the micropore chambers were recovered. Contents of each chamber were emptied and larvae were counted and examined microscopically for adherence of cells and for larval death. Larvae that were transparent, straight, and with no movement were counted as dead. Larvae that were active, coiled, and translucent were counted as live. The percentage of protection was expressed as the number of dead parasites/number of total parasites recovered × 100.

### Levels of Secreted Cytokines in Culture Supernatant of Splenocytes

Single cell suspension of spleen cells was prepared and stimulated with 1 µg/ml of r*Bm*HAXT or ConA. Unstimulated spleen cells were kept as negative control for the assay. After 72 h incubation, culture supernatants were collected and levels of IL-2, IL-4, IL-6, IFNγ, TNFα, IL-10, and IL-17A were determined using a cytokine bead array kit (BD Bio Sciences, San Jose, CA, USA).

### Analysis of T Cell Subsets by Flow Cytometer

Spleen cells from the above cultures were then washed and labeled with fluorescent-labeled anti-mouse CD3 (APC), CD4 (PE), and CD8 (PE/cya7) and the percent population of each cell type was determined in a flow cytometer. Briefly, cells were incubated with FcγII receptor blocker in staining buffer (2% FBS + 0.1% sodium azide) for 30 min at 4°C with subsequent wash in staining buffer. All the three fluorescent-labeled antibodies were added to the cells and incubated for 1 h at 4°C in the dark. After washing with staining buffer, cells were fixed in 4% paraformaldehyde and analyzed in a BD Facs Calibur (BD Biosciences) flow cytometer.

Another set of cells from the above experiment was stained with CD3 (APC) and within the CD3 gated population, the CD62L (PE/Cya7) and CCR7 (PE) positive T cells were identified as T-central memory cells. We also stained these cell populations for intracellular IFN-γ (FITC) to determine the percent of IFN-γ positive T-central memory cells.

## Statistical Analysis

Data presented are mean ± SD. Statistical significance of mean differences among different sample groups was analyzed using non-parametric Kruskal–Wallis test followed by Bonferroni correction for multiple tests using SPSS software (v24.0, IBM, NY). The significance level was defined as *p* < *0.05*.

## Results

### *rBm*HAXT Was Cloned and Purified

**Figure 1:**
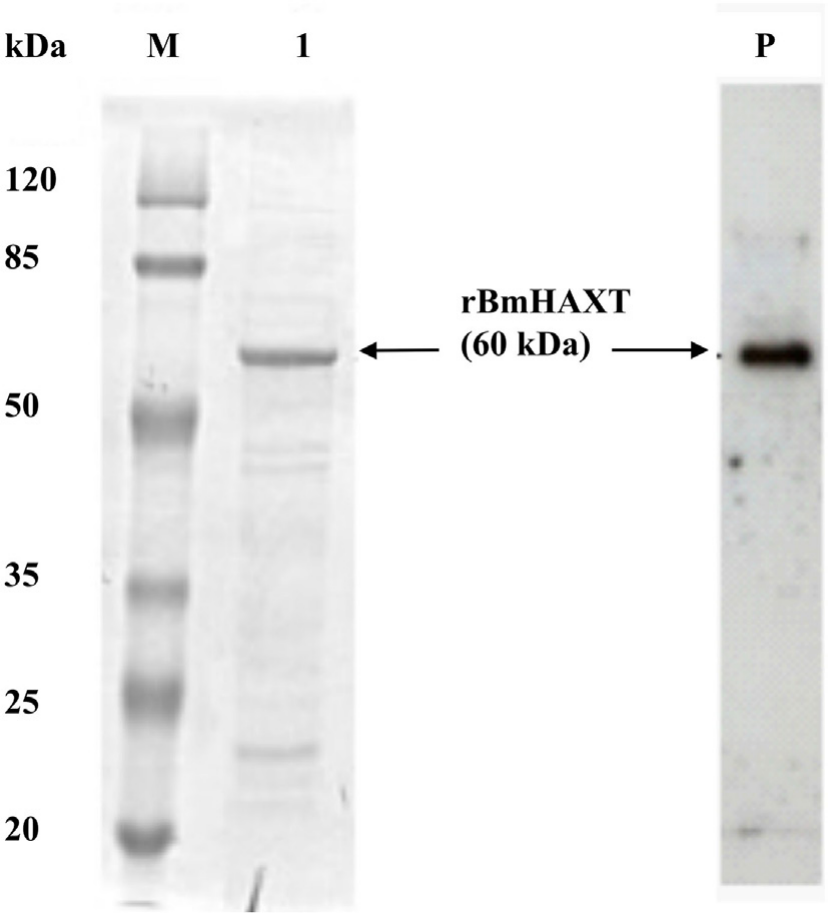
Expression of recombinant proteins. Analysis of expressed and purified r*Bm*HAXT protein was carried out using 12% SDS-PAGE gel. Lanes: M, protein molecular weight marker; 1, r*Bm*HAXT purified. Lane P: Western blot analysis of purified r*Bm*HAXT showing 6× his-tagged protein.

We cloned and prepared purified recombinant *Bm*HAXT protein. On the SDS-PAGE gel, the molecular mass of r*Bm*HAXT was around 60 kDa and appeared as a single band (Figure 1). Endotoxin levels in the final purified preparations were <3 EU/0.1 mg of protein.

### Titer of r*Bm*HAXT-Specific IgG Antibody Was High in the Serum and Peritoneal Fluid of All Immunized Mice

**Figure 2:**
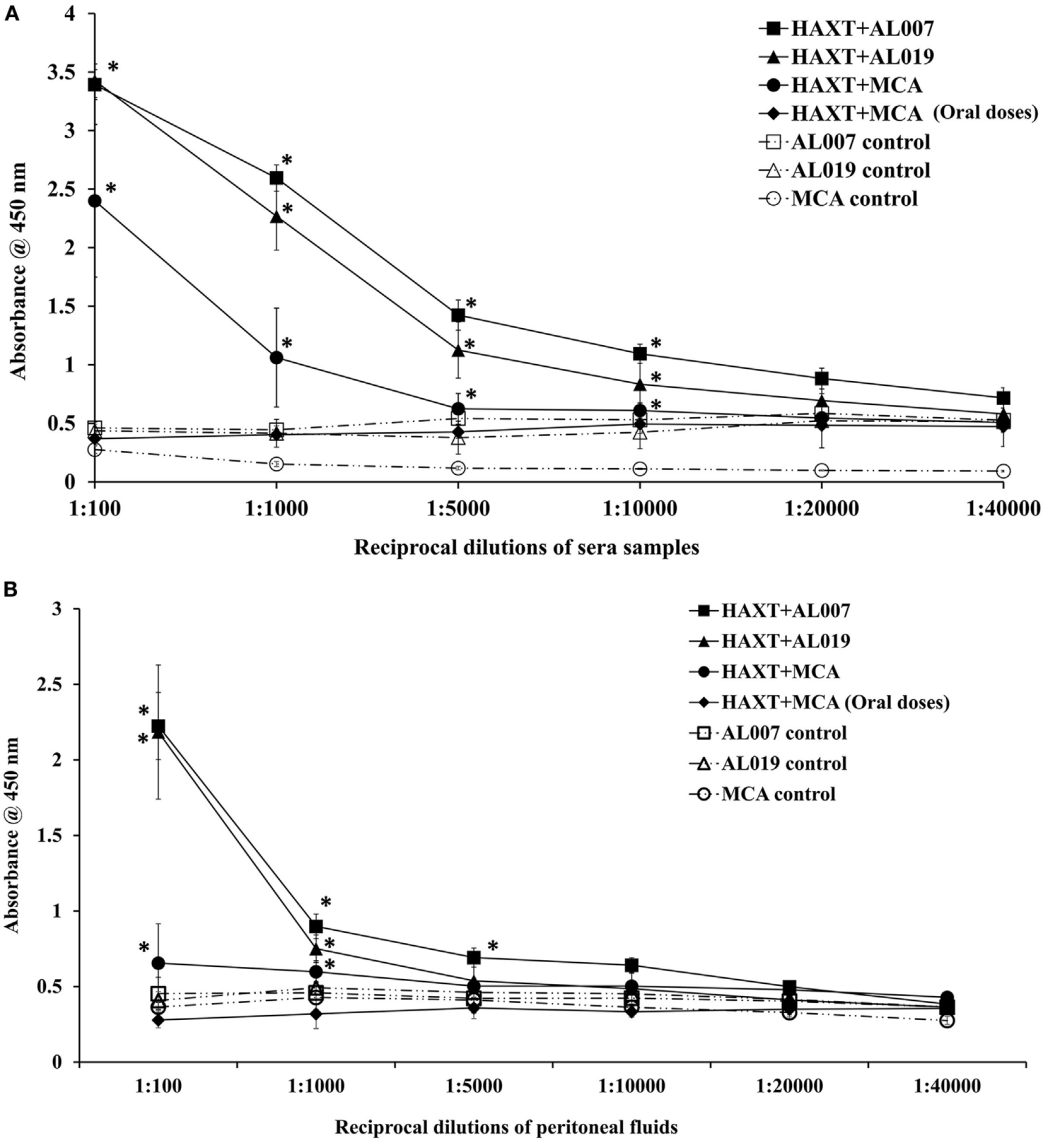
Titer of r*Bm*HAXT-specific IgG antibodies in immunized animals. **(A)** r*Bm*HAXT-specific IgG titer in sera samples showed that both AL007 and AL019 adjuvants-based formulations are better in generating the IgG titer (1:20,000) when compared to mannosylated chitosan (MCA) formulations (1:10,000). Pre-immune sera samples were used as baseline controls in these assays. **(B)** In peritoneal fluid, titer of IgG is comparatively low (1:10,000) in AL007 and AL019-based formulations when compared to sera samples. In contrast, IgG titer is significantly reduced in r*Bm*HAXT + MCA-treated animal’s peritoneal fluid than sera samples. *n* = *5* per group, *p < 0.05 compared to respective group as analyzed by Kruskal–Wallis test followed by Bonferroni correction for multiple analysis.

The titer of r*Bm*HAXT-specific IgG antibody was high (1:20,000) in r*Bm*HAXT + AL007 group and in r*Bm*HAXT + AL019 group (*p* < 0.05) (Figure 2A). However, the titer was less (1:10,000) in r*Bm*HAXT + MCA group. In r*Bm*HAXT + MCA group where all the doses were given orally, we found there was very little titer of antigen-specific antibodies nearly same as AL007, AL019, and MCA adjuvant control groups (Figure 2A). Similarly, when we analyzed the peritoneal fluids, we observed high titer of antigen-specific IgG antibody in r*Bm*HAXT + AL019 and *rBm*HAXT + AL007-vaccinated animals when compared to *rBm*HAXT + MCA group (Figure 2B). The titer of IgG antibodies was less in the peritoneal fluids when compared to the respective sera samples from the same animals.

### Antibody Isotypes in Serum and Peritoneal Fluid

**Figure 3:**
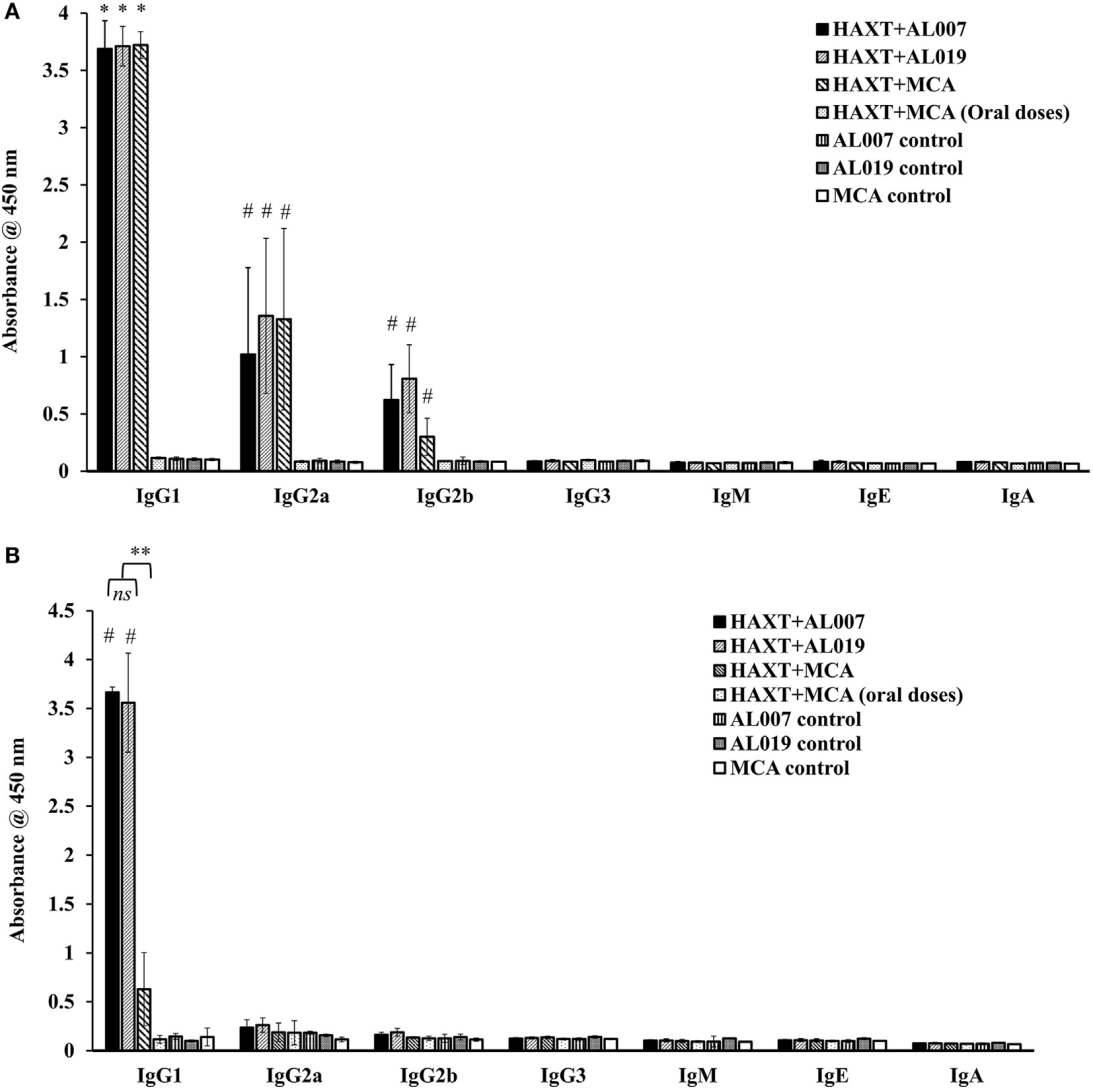
Level of antigen-specific antibody isotypes was determined by indirect ELISA in sera samples as well as in peritoneal fluid. **(A)** IgG1 was found predominant in all the immunized animals when compared to their respective adjuvant control groups. Specifically, IgG2a was found to be increased in r*Bm*HAXT + AL019 and r*Bm*HAXT + mannosylated chitosan (MCA) groups compared to the r*Bm*HAXT + AL007 group. IgG2b antibody level was significantly increased in r*Bm*HAXT + AL019 immunized group **(B)** IgG1 is much lesser in the peritoneal fluid of r*Bm*HAXT + MCA (s/c) group when compared to r*Bm*HAXT + AL007 and r*Bm*HAXT + AL019 groups. Moreover, IgG2a and IgG2b antibodies were not detected in peritoneal fluid of all the group of animals as observed in the sera of vaccinated animals. *n* = *5* per group, *p < 0.05, ^#^*p* < 0.001 compared to respective control group. **p < 0.001 compared to r*Bm*HAXT + MCA group. ns = not significant, as analyzed by Kruskal-Wallis test followed by Bonferroni correction for multiple analysis.

To determine the type of humoral immune response generated against *rBm*HAXT, we determined the antibody isotypes IgG1, IgG2a, IgG2b, IgG3, IgE, IgM, and IgA in serum and peritoneal fluid samples. Our results showed that, IgG1 was the predominant isotype of antibody in all vaccinated groups (*p* = 0.0001) except *rBm*HAXT + MCA (all oral dose) group which was similar to the controls (Figure 3A). Titer of IgG2a and IgG2b antibodies were also significantly high in all vaccinated animals (*p* < 0.05) compared to controls except in *rBm*HAXT + oral MCA-vaccinated group (Figure 3A). Titer of IgG3, IgE, IgM, and IgA antibodies in the vaccinated animals did not show any significant changes compared to the controls. Significantly, high titers of IgG1 antibodies were present in the peritoneal fluids of r*Bm*HAXT + AL007 and *rBm*HAXT + AL019-vaccinated animals (*p* = 0.0001) compared to *rBm*HAXT + MCA subcutaneous group and controls (Figure 3B). Titer of IgG2a and IgG2b antibodies in the peritoneal fluid of all vaccinated animals were not significantly different from the controls (Figure 3B).

### Vaccination With r*Bm*HAXT + AL019 Conferred Maximum Protection

**Figure 4:**
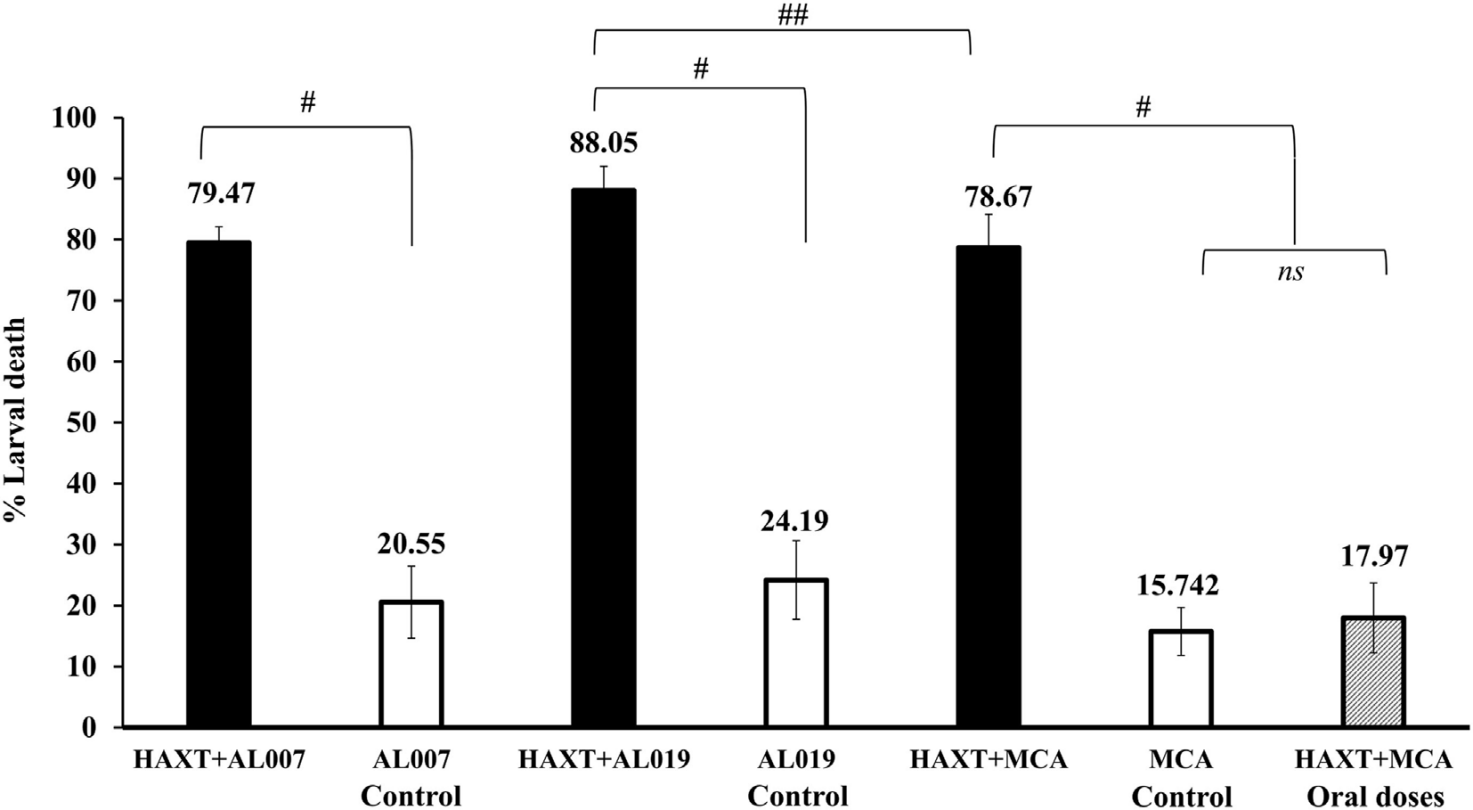
Vaccine-induced protection was calculated by percent larval death in immunized animals. Approximately, 20 live L3s were sealed in micropore chambers and surgically implanted in peritoneal cavity of mice. After 72 h, micropore chambers were taken out and L3s larvae were recovered and counted as live and dead worms to determine the percent larval death. Compared to adjuvant control groups, there was significant death of larva in vaccinated groups. Results showed that r*Bm*HAXT + AL019 immunized group showed maximum protection (88%), followed by 79.47 and 78.67% in *rBm*HAXT + AL007 and *rBm*HAXT + mannosylated chitosan, respectively. *n* = 5 mice per group, ^#^*p* < 0.0001 and ^##^*p* < 0.05, ns = not significant compared to respective group as analyzed by Kruskal–Wallis test followed by Bonferroni correction for multiple analysis.

Vaccine-induced protection was determined using a micropore chamber challenge method as described previously (28–33). Our results showed that maximum protection was observed in animals vaccinated with *rBm*HAXT + AL019 (88.05 ± 3.9%; *p* = 0.0001) followed by *rBm*HAXT + AL007 (79.47 ± 2.6%; *p* = 0.0001) and *rBm*HAXT + MCA (78.67 ± 5.47%; *p* = 0.0001) (Figure 4). Larvae collected from micropore chamber were observed under the light microscope and showed several cells were found attached to the surface of the dead larvae. These results suggest that AL019 may be a better adjuvant for *rBm*HAXT compared to AL007 (*p* = 0.0037) and MCA (*p* = 0.02). Vaccination with *rBm*HAXT + oral MCA conferred only 17.97 ± 5.75%, which was similar to the adjuvant control groups (Figure 4).

### Spleen Cells From r*Bm*HAXT-Vaccinated Animals Secreted Both Th1 and Th2 Cytokines

**Figure 5:**
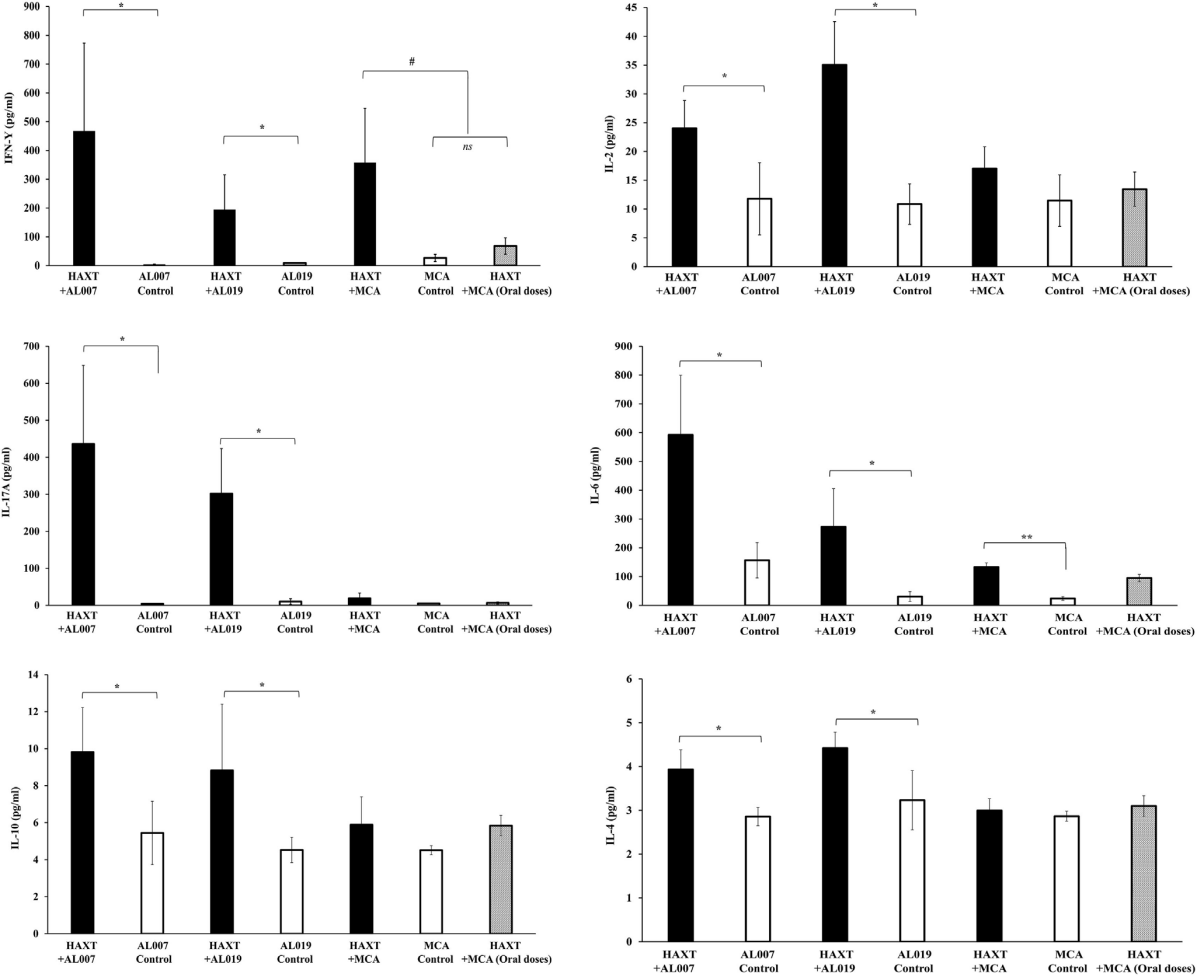
Level of cytokines (IFN-γ, IL-2, IL-6, IL-17A, IL-4, and IL-10) in culture supernatant of spleen cells were determined by cytokine bead array. After 72 h incubation, culture supernatant was collected and stored in −80°C until use. 50 µl of supernatant was used in the assay as per the manufacturer recommendation. Results showed that r*Bm*HAXT + AL007 and r*Bm*HAXT + AL019 were better in the expression of both Th1/Th2 cytokines compared to *rBm*HAXT + mannosylated chitosan group. This showed that AL007 and AL019 can produce balanced Th1/Th2 cytokines response. *p < 0.05, ^#^*p* < 0.01, **p < 0.001 compared to respective group, ns = not significant as analyzed by Kruskal-Wallis test followed by Bonferroni correction for multiple analysis.

Cytokines level in the culture supernatants of spleen cells was determined using a cytokine bead array. Our results showed that secreted levels of Th1 (IFN-γ, IL-2, IL-6, and IL-17A) and Th2 (IL-4 and IL-10) cytokines were significantly (*p* < 0.05) increased in the culture supernatants of spleen cells from *rBm*HAXT + AL019 and *rBm*HAXT + AL007-vaccinated animals compared to respective adjuvant control animals (Figure 5). Spleen culture supernatants from *rBm*HAXT + MCA-vaccinated animals had significantly high levels of IFN-γ (*p* = 0.01) and IL-6 (*p* = 0.0001) (Figure 5) compared to the respective adjuvant control. However, there was no significant difference in the levels of the other cytokines measured. Cytokine levels in the culture supernatants from *rBm*HAXT + oral MCA group were similar to the adjuvant controls (Figure 5).

### T_CM_ Cells Were Generated in the Spleen of r*Bm*HAXT-Vaccinated Animals

**Figure 6:**
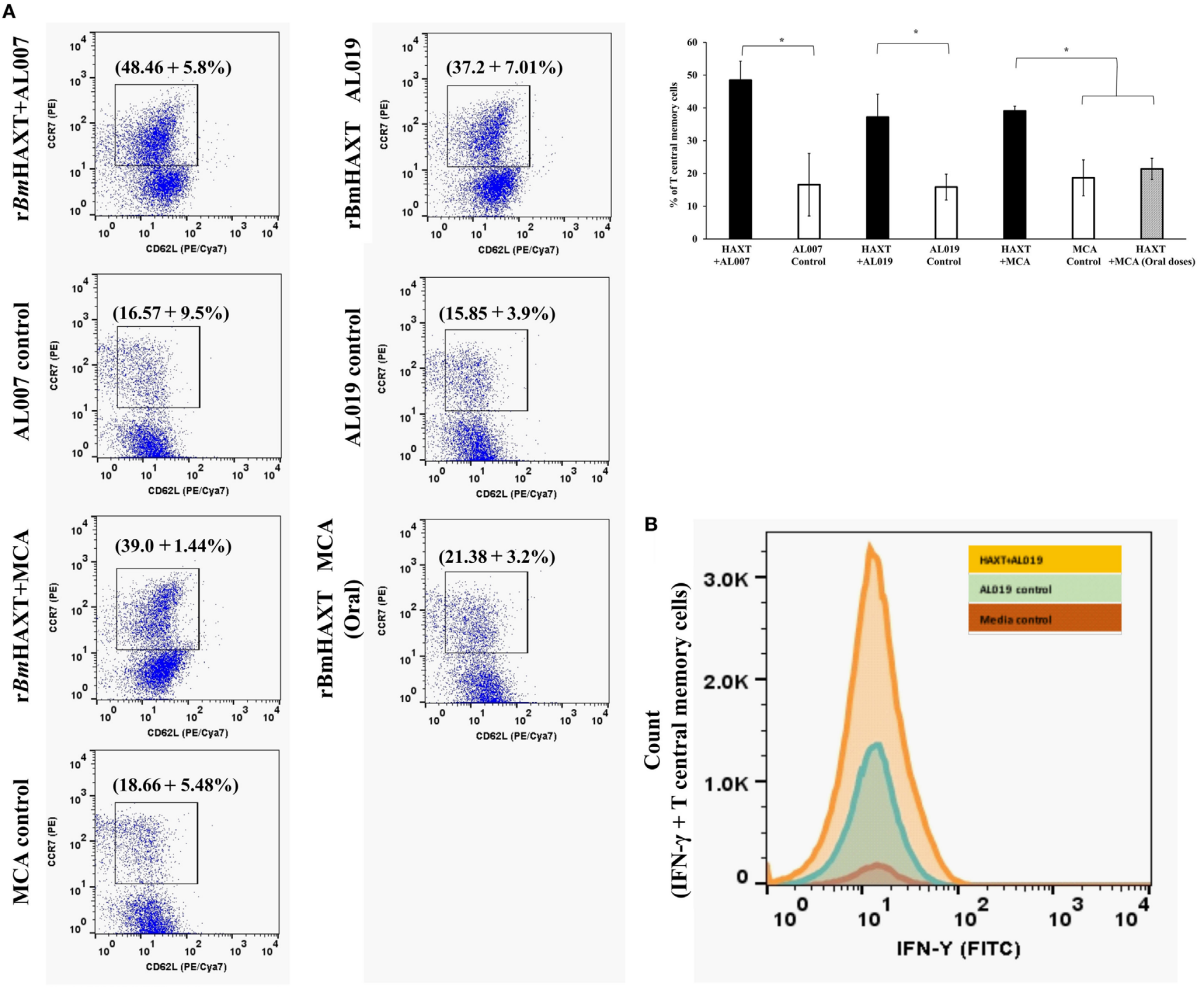
T-central memory cells and their IFN-γ secretion in the spleen of immunized mice. **(A)** Cultured spleen cells were stained with anti-mouse CCR7 (PE) and CD62L (PE/Cya7) antibodies. Cells which were dual positive for both the markers were selected as T central memory cells. Results showed that, T central memory cells were significantly high in r*Bm*HAXT + AL007 compared to all the groups. Similarly, r*Bm*HAXT + mannosylated chitosan (MCA) and r*Bm*HAXT + AL019-treated mice also showed significant increase in T central memory cells. **(B)** Histogram showed that r*Bm*HAXT + AL019 formulation-treated animals showed significantly increased expression of IFN-γ in T central memory cells. However, no significant difference was found in r*Bm*HAXT plus Al007/MCA-treated groups with respect to their respective control group. *p < 0.05 compared to respective group as analyzed by Kruskal-Wallis test followed by Bonferroni correction for multiple analysis.

Spleen cells were cultured at 37°C for 72 h, stimulated with 1 µg/ml of r*Bm*HAXT protein. After 72 h, cells were harvested and stained with CD3/CD4/CD8 antibodies and evaluated in flow cytometer. There was a slight but significant increase in the CD8+ cell population in r*Bm*HAXT + AL019-treated group (*p* ≤ 0.05) compared to the other groups (data not shown). To determine the percent of T_CM_ cells in the spleen, splenocytes were stained with CD62L/CCR7 antibodies and analyzed in a flow cytometer. Cells that were dual positive for CD62L/CCR7 were considered as T_CM_ cells. Our results showed that r*Bm*HAXT-treated animals showed high percentage of T_CM_ cells irrespective of the adjuvant used (*p* ≤ 0.001) (Figure 6A).

### T_CM_ Cells Were Predominantly IFNγ+

IFN-γ secreting T_CM_ cells are believed to play a major role in vaccine-induced protection in parasitic infections (44). Therefore, we measured the percentage of CD62L+ CCR7+ T_CM_ cells that expressed intracellular IFN-γ. Our results show that cells from r*Bm*HAXT + AL019-vaccinated animals had the significantly (*p* < 0.01) high percentage of IFN-γ+ T_CM_ cells compared to r*Bm*HAXT + AL007 and r*Bm*HAXT + MCA-vaccinated groups (Figure 6B).

## Discussion

Results presented in this study show that both AL007 (alum) and AL019 (GLA + alum) are excellent adjuvants for r*Bm*HAXT immunization in mice. Both the adjuvants elicited significant immunogenicity in vaccinated mice. However, the vaccine-induced protection was higher in animals immunized with r*Bm*HAXT + AL019 suggesting that AL019 is probably a better adjuvant for r*Bm*HAXT immunizations than alum or MCA.

Our previous studies with a trivalent vaccine (r*Bm*HAT) given along with alum (AL007) as the adjuvant gave close to 95% protection in the mouse model (29–33), however, when we tested the formulation in macaques only modest protection (~35%) was achieved against *B. malayi* L3 challenge infections (34). This prompted us to search for an improved vaccine formulation that could be eventually used in macaques. Therefore, in this study we used the mouse model to initially evaluate and dissect out the immune responses. In an effort to improve the current trivalent vaccine, the first modification we made was to add another vaccine candidate thioredoxin peroxide (r*Bm*TPX-2) to the trivalent formulation.

Thioredoxin peroxidase is a member of the peroxidoxin superfamily of proteins that play a key role in a variety of cellular processes, such as DNA synthesis, defense against oxidative stress, detoxification, protein folding, and repair and operate through redox cascades that involve transfer of reducing equivalents from NADPH to targets through reversible dithiol-disulfide reactions (45). TPX is expressed in several organisms, including helminths (46–52) and protozoan (53) parasites. One of the major functions of TPX-2 in parasites is to protect the parasites from damaging effects of host-generated oxidative stress by producing H_2_O_2_-detoxifying activity (50, 54). TPX-2 protein is predominantly localized to the surface of the helminth parasites mainly hypodermis and cuticle (50, 54). In *Onchocerca volvulus* the Ov-tpx-2 cDNA represents roughly 2.5% of the total cDNAs from the L3 cDNA library suggesting that these are important proteins for the parasite helping them to evade host defense mechanisms (51). Given its critical role for survival of the parasite in the host, TPX-2 is a potential target for vaccine development. Our group and others (24, 27, 28, 40–42) have previously reported the vaccine potential of TPX. Studies showed that r*Bm*TRX could confer 62% protection against *B. malayi* challenge in *Mastomys* model (43) and 43–69.5% protection in mouse and jird models (27, 28).

We inserted the gene sequence of *Bmtpx2* in between ALT-2 and TSP. This allowed us to use the same primers as the trivalent gene to amplify the tetravalent gene. Subsequent expression of the tetravalent vaccine protein (r*Bm*HAXT) showed that we could get substantially higher and better expression of the tetravalent protein compared to our previous trivalent protein preparations. As demonstrated in Figure 1, we could get >98% pure final r*Bm*HAXT vaccine protein in our studies.

Analysis of the type of immune responses generated following TPX-2 immunization suggested that predominantly an IFN-γ-mediated response is generated in the mouse model (24, 27, 28, 40–42). Our previous studies using the trivalent formulation (r*Bm*HAT plus alum) in rhesus macaque showed that a Th2 biased response was predominant with a weak IFN-γ response, which we believed was the reason for poor vaccine-induced protection in macaques (34). Thus, in the current study we hypothesized that including *Bm*TPX-2 as an additional vaccine antigen could improve the Th1 response after vaccination. Our results confirmed this notion. The tetravalent formulation was found to significantly increase the levels of IFN-γ-producing antigen-specific memory cells in vaccinated animals irrespective of the adjuvants tested. Further evidence comes from the analysis of the spleen cells, where the spleen cells from r*Bm*HAXT-vaccinated animals were shown to secrete IL-4, IL-10, and IFN-γ in response to r*Bm*HAXT suggesting the generation of a balanced Th1/Th2 response. There was also an increase in IL-17-secreting cells in r*Bm*HAXT-vaccinated animals. IL-17 has been shown to be critical for promoting vaccine-induced protection in several systems (55–61).

One of our recent studies suggested that including GLA-SE/alum (a synthetic toll-like receptor 4 agonist on alhydrogel, AL019) as an adjuvant along with the trivalent formulation (r*Bm*HAT) promoted a balanced Th1/Th2 response in the mouse model and gave better protection (30). GLA-SE is shown to be an excellent adjuvant for several human vaccine formulations (62, 63) and other helminth vaccine formulations (64). Therefore, in this study we evaluated if AL019 is a better adjuvant for the tetravalent (r*Bm*HAXT) formulation. Our results showed that including AL019 as an adjuvant along with r*Bm*HAXT gave significantly better protection (~88%) than alum as adjuvant that gave slightly less (79%) protection rate.

Similarly, we also tested another adjuvant formulation, MCA. One of our recent studies showed that MCA is a potential adjuvant for the trivalent (r*Bm*HAT) formulation conferring close to 88% protection in mice (30). However, in this study when we used MCA as an adjuvant for the tetravalent formulation, the protection rate was slightly low (79% protection) but significant. One of the reasons for testing MCA as an adjuvant in this study is because of its potential to support the vaccine as an oral delivery platform, which may be advantageous for clinical use. Nevertheless, results from this study showed that MCA is as good as alum in generating the protective immune responses, especially when the first dose of MCA plus the vaccine antigen was given subcutaneously and the subsequent booster doses were given orally. However, when all the MCA plus vaccine antigen doses (prime and booster doses) were given orally, MCA was not as effective as an adjuvant for the r*Bm*HAXT immunizations. Thus, our results showed that AL019 may be a better adjuvant for r*Bm*HAXT vaccine formulation compared to alum and MCA.

Analysis of the titer of IgG antibodies in the serum and peritoneal fluids showed that alum and AL019 elicited the maximum titers of IgG antibodies compared to MCA adjuvant. Similarly, all three adjuvants elicited a balanced Th1/Th2 antibody response (IgG1, IgG2a, and IgG2b). These findings are similar to our previous report [30] and correlated well with the protective responses observed in this study. r*Bm*HAXT vaccination did not elicit any IgE responses as was reported before for the r*Bm*HAT vaccination in rhesus macaque (34) and mice (30). Surprisingly, oral delivery of vaccine antigen along with MCA (prime and booster doses) did not yield any antibody responses in the serum or peritoneal fluids suggesting that oral route alone may not be a viable vaccine delivery approach for r*Bm*HAXT + MCA vaccination.

In conclusion, our present study show that AL019 is a better adjuvant formulation for *rBm*HAXT vaccination in the mouse model, based on the higher rate of protection and the strong immune responses generated following immunization. Further studies need to be performed in the non-human primate models with the tetravalent formulation plus AL019.

## Ethics Statement

Use of mice and the experimental procedures performed in this study were reviewed and approved by the IACUC committee at the University of Illinois College of Medicine at Rockford.

## Author Contributions

NC, VK, and RK planned the experiments. NC, VK, and RK interpreted the results and analyzed the data. NC, VK, and RK performed immunization and challenge experiments. NC, PB, and VK performed the flow cytometry and proliferation assays. VK performed the ADCC and MPO assays. NC, PB, and VK performed all the cell-based assays. All authors contributed to the writing of the manuscript.

## Conflict of Interest Statement

The authors declare that the research was conducted in the absence of any commercial or financial relationships that could be construed as a potential conflict of interest.
